# *Alpinia oxyphylla* Miq. Extract Prevents Diabetes in Mice by Modulating Gut Microbiota

**DOI:** 10.1155/2018/4230590

**Published:** 2018-06-04

**Authors:** Yiqiang Xie, Man Xiao, Yali Ni, Shangfei Jiang, Guizhu Feng, Shenggang Sang, Guankui Du

**Affiliations:** ^1^First Affiliated Hospital of Hainan Medical University, Haikou 571199, China; ^2^Department of Biochemistry and Molecular Biology, Hainan Medical University, Haikou 571101, China

## Abstract

Recently, the role of gut microbiota in the development of obesity and type 2 diabetes mellitus (T2DM) has been highlighted. We performed an 8-week administration protocol on T2DM (C57BL/6J db-/db-) mice and fecal samples were collected. Comparisons of fecal bacterial communities were performed between db-/db- mice and normal mice (DB/DB) and between the db-/db mice treated and untreated with AOE using next-generation sequencing technology. Our results showed that the db-/db-AOE group had improved glycemic control and renal function compared with the db-/db-H_2_O group. Compared with the db-/db-H_2_O group, AOE administration resulted in significantly increased ratio of Bacteroidetes-to-Firmicutes in db-/db- mice. In addition, the abundance of *Akkermansia* was significantly increased, while *Helicobacter* was significantly suppressed in the db-/db-AOE group compared with the db-/db-H_2_O group. Our data suggest that AOE treatment decreased blood glucose levels and significantly reduced damage of renal pathology in the T2DM mice by modulating gut microbiota composition.

## 1. Introduction

Diabetes mellitus (DM) is a metabolic disease whose principal cause is obesity-linked insulin resistance. This disease is becoming a major public health challenge, and the incidence of DM is projected to rise to 439 million by 2030, while 90% patients have type 2 diabetes mellitus (T2DM) [1]. The main clinical methods in DM therapy have involved alleviating hyperglycemia, hypertension, and dyslipidemia [2]. A mutation in the leptin receptor gene in db/db mice is a rodent model of genetic diabetes, and the development of glomerular hypertrophy is evident early in response to hyperglycemia [3].

In recent years, the role of gut microbiota in host physiology, disease, and behavior has been generally recognized [4]. Next-generation sequencing technology provides a new perspective of the role of microbiota in human health, including shaping our immune system, metabolism, and energy balance [5]. Altered gut microbiota is related to multiple diseases, such as T2DM and obesity [5]. Recently, growing evidence has shown that gut microbiota during clinical T2DM results in compositional changes between patients and healthy controls, including an obesity-related change in the abundance ratio of Bacteroidetes : Firmicutes [6, 7] and a decreased abundance of mucin-degrading *Akkermansia muciniphila* in overweight children and pregnant women [8]. Notably, reduced levels of *Akkermansia* have been observed in patients with inflammatory bowel diseases and metabolic disorders [9]. It has been reported that fruits and vegetables, such as bamboo shoot fiber [10], capsaicin [11], and polyphenol [12, 13], the consumption of which is strongly associated with a healthy lifestyle, prevented diet-induced obesity in association with a remarkable change of gut microbiota, especially increase in the abundance of *Akkermansia* [14, 15]. Therefore, understanding the alteration of gut microbiota under different situations will be beneficial for the diagnosis and treatment of different diseases.

*Alpinia oxyphylla* (*A. oxyphylla*) can be used for different purposes. In China, *A. oxyphylla* is traditionally used as a dietary supplement. The herb is used in traditional Chinese medicine and has been widely used to treat diarrhea, abdominal pain, intestinal disorders, dementia, and inflammatory conditions [16–18]. Component isolates from *A. oxyphylla* show antioxidative stress properties, such as protocatechuic acid [19, 20]. Previous studies have shown that *A. oxyphylla* can promote the migration and proliferation of human adipose tissue-derived stromal cells [21, 22]. In our previous study, we found that *A. oxyphylla* extract (AOE) exhibits an antioxidant and anti-T2DM capacity [23, 24]. However, the mechanism of AOE-mediated anti-T2DM function remains largely unknown. Moreover, because of the importance of gut microbiota in the development of T2DM and obesity, we investigated the alteration of gut microbiota in db-/db- mice treated with AOE.

## 2. Material and Methods

### 2.1. Preparation of the Plant Extract

The ripe fruits of *A. oxyphylla* were collected from herb market (Haikou, China) and were identified by Dr. Qiang Liu (Department of Pharmacognosy, Hainan Medical College). Briefly, the ripe fruits were extracted with water for 16 hours at 90°C two times, and then the extract was lyophilized. The dry yield was 8% (*w*/*w*).

### 2.2. Animals

For this study, we performed experiments strictly in accordance with protocols approved by the Ethics Committee of Hainan Medical College for Animal Care and Use. The animals were monitored twice per week, and none of the animals showed severe illness, died, or appeared moribund during any of the experiments.

All mice purchased from the Model Animal Research Center of Nanjing University, China. Mice were housed in an environmentally controlled room at 22 ± 1°C with a relative humidity of 50 ± 5% under a light cycle of 12 h light/12 h dark. Specific pathogen-free hygienic status was approved by a health certificate according to the Federation of European Laboratory Animal Science Associations guidelines [25]. They had free access to standard rodent diet (GMLAC, Guangdong) and water, unless stated otherwise in substudies. Mice were maintained in polycarbonate individually ventilated cages with corncob bedding (GMLAC, Guangdong). Body weights were monitored weekly during the studies.

A total of 30 male mice (3 to 4 weeks old), including 6 DB/DB mice and 24 db-/db- mice (i.e., mice carrying a mutation in the leptin receptor gene) with a C57BL/Ks background (BKS.Cg-Dock7^m+/+^Lepr^db^/Nju, NBRI 2B051), were divided into 5 groups, with 6 animals in each group. Mice were allowed to acclimatize for 1 week before experiment. The DB/DB mice group and db-/db-H_2_O group were given saline only, and the AOE-treated group received 100 mg/kg (db-/db-AOE100), 300 mg/kg (db-/db-AOE300), or 500 mg/kg (db-/db-AOE500) of AOE via the intragastric route once a day for 8 weeks (approximately, 0.2 mL in volume), respectively.

At 8th week, 24 h urine collection and feces were obtained. Then, the mice were euthanized by chloral hydrate anesthesia. Blood and kidney samples were taken for further analysis. Blood samples were collected from the hepatic portal vein into tubes containing EDTA anticoagulation and centrifuged (3000 rpm for 15 minutes at 4°C) for separating the plasma, which was then frozen at −70°C for biochemical analysis.

### 2.3. Measurement of Concentration of Glucose and Albumin

Glucose was measured using commercial kits (glucose (F006)) (Nanjing Jiancheng Bioengineering Institute, Nanjing, China), according to the manufacturer's instructions. Glucose concentration was assayed by the glucose oxidase method [26]. HbA1c levels were evaluated using commercial kits (Beijing HOMA Biologicals, China). Urine albumin was measured using an Albumin Mouse ELISA kit (ab108792), which was purchased from Abcam (Cambridge, UK). The absorbance was read using an automated microplate ELISA reader (BioTek Instruments Inc., Winooski, VT, USA), and concentrations were calculated by the standard curve run on each assay plate. All samples were measured in duplicate.

### 2.4. Glucose Tolerance Test

The oral glucose tolerance test (OGTT) was performed after 6 weeks of AOE administration. After an overnight fast, the animals were administered glucose (1.0 g/kg) solution orally. After 0, 30, 60, 90, and 120 minutes, Blood samples were collected via the tail vein and measured for blood glucose concentration.

### 2.5. Histological Examination

For histological analysis, animals were perfused with phosphate-buffered saline and 4% paraformaldehyde in phosphate-buffered saline. Organs were dissected and kept in the same fixation solution overnight at 4°C. Samples were embedded in paraffin following dehydration in ethanol. Tissues were cut into 6 *μ*m sections and stored for staining. For hematoxylin-eosin (Sigma) staining, sections were freed of paraffin and stained. The slides were evaluated by blinded visual inspection including an experienced pathologist. All biopsies were analyzed quantitatively to determine the percentages of glomeruli with (1) segmental and global sclerosis, (2) mesangial cell proliferation, and/or (3) increase in mesangial matrix. All biopsies were scored semiquantitatively for the percentages of the aforementioned lesioned glomeruli (score 0, 0%; score 1, 1 to 24%; score 2, 25 to 49%; and score 3, ≥50% of all glomeruli) [27]. Scoring of the chronic tubulointerstitial injury was based on the percentage of tubular atrophy and interstitial fibrosis and was graded as mild (1+) if involving <25%, moderate (2+) if involving 25–50%, and severe (3+) if involving ≥50%.

### 2.6. Microbiota Analysis by 16S rRNA Gene Sequencing

Total fecal DNA was extracted by QIAamp DNA Stool mini kit (Qiagen, Hilden, Germany) according to the manufacturer's instructions. The V3-V4 region (~450 bp) of the 16S rRNA gene was amplified using the universal primers 341F (5′-CCTACGGGNGGCWGCAG-3′) and 802R (5′-TACNVGGGTATCTAATCC-3′). The forward primer 341F contained the sequence of the Roche B adapter for Illumina library construction and the reverse primer 802R attached to the Roche A adapter. Sequencing was carried out on Illumina HiSeq/MiSeq platform, and at least 48,000 reads per sample (mean reads per sample 65,610 ± 1346) was obtained. FLASH [28] and QIIME (Quantitative Insights Into Microbial Ecology) was used to merge reads. Greengenes database [29] and the UPARSE algorithm [30] were used to group paired-end joined sequences into operational taxonomic units (OTUs) (97% threshold of pairwise identity). The RDP classifier [30] was used to annotate taxonomic information. Alpha diversity (within samples) and beta diversity (among samples) were analyzed by in-house Perl scripts. Unweighted UniFrac for principal coordinate analysis (PCoA) was used to assess the variation between experimental groups.

### 2.7. Statistics

All data are presented as the mean ± SEM. Statistical analysis of physiological and biochemical data was conducted using the GraphPad Prism version 7.0 (GraphPad Software, San Diego, CA). One-way ANOVA followed by Tukey's test was used to determine significant mean differences. Data was expressed as mean ± SE. *P* values less than 0.05 were considered statistically significant. A two-way nonparametric permutation analysis of variance (perMANOVA) was applied to evaluate the bacterial differences under different treatments. The *P* value from Fisher's exact test was corrected by the Holm-Bonferroni method. Canonical correspondence analysis (CCA) was performed using Canoco software version 4.5 [31].

## 3. Results

**Figure 1:**
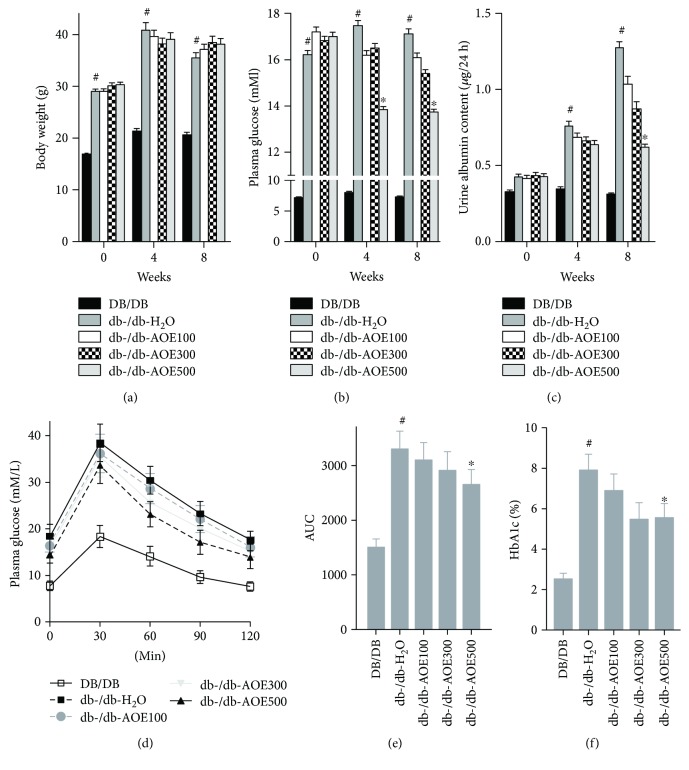
Effects of AOE on body weight, blood glucose levels, and renal function. The alteration of body weight (a), blood glucose (b), and urine albumin (c) after AOE treatment for 4 and 8 weeks. ^∗^*P* < 0.05 for the db-/db-AOE group compared with the db-/db-H_2_O group; ^#^*P* < 0.05 for db-/db-H_2_O-treated compared with DB/DB.

After AOE administration, changes in body weight of db-/db- mice did not differ (Figure 1(a)), while plasma glucose significantly decreased (AOE administration for 4 weeks, 13.2% (*P* = 0.0271) in db-/db-AOE100, 10.6% (*P* = 0.0143) in db-/db-AOE300, and 29.3% (*P* = 0.0048) in db-/db-AOE500) compared with the db-/db-H_2_O group in a dose-dependent manner (Figure 1(b)). Meanwhile, urine albumin excretion decreased significantly after AOE administration (23.1% (*P* = 0.0176) in db-/db-AOE100, 35.1% (*P* = 0.0077) in db-/db-AOE300, and 52.9% (*P* = 0.0052) in db-/db-AOE500) for 8 weeks compared with the db-/db-H_2_O group in a dose-dependent manner (Figure 1(c)). The effects of the AOE on the glucose intolerance were measured. In oral glucose tolerance test, the db-/db- mice treated with AOE had improved glucose intolerance compared to db-/db-H_2_O mice, in which the db-/db-AOE500 mice revealed the lower plasma glucose concentrations and lower area under the curve (AUC) of glucose (19.9%, *P* = 0.0386) (Figures 1(d), 1(e)). Glycated hemoglobin, HbA1c, was tested at the end of AOE treatment. Compared to DB/DB mice, db-/db-H_2_O mice displayed elevated HbA1c levels (3.2 fold, *P* = 0.0044). On the 8 weeks of treatment, 300 mg/kg (30.9%, *P* = 0.0076) and 500 mg/kg AOE (30.6%, *P* = 0.0082) both reduced HbA1c levels significantly, indicating efficient glycemic control (Figure 1(f)). Those results showed that 500 mg/kg AOE could effectively decrease blood glucose level and urine albumin excretion.

**Figure 2:**
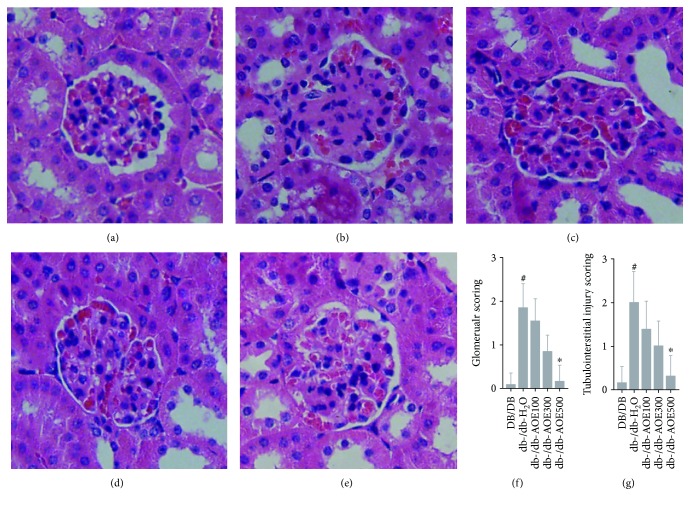
HE staining in the kidney of mice after 8 weeks of AOE treatment. Representative HE staining showed the inflammatory cell infiltration in the kidney of DB/DB mice (a), db-/db-H_2_O (b), db-/db-AOE100 (c), db-/db-AOE300 (d), and db-/db-AOE500 (e). Bar graphs (f) showed the changes of average infiltration percentage of inflammatory cells in the kidney. Data represent the mean ± SD (*n* = 6). ^∗^*P* < 0.05 for the db-/db-AOE group compared with the db-/db-H_2_O group, ^#^*P* < 0.05 for db-/db-H_2_O-treated compared with DB/DB.

H&E-stained kidney tissue sections were used to assess renal morphology and pathology. An example of normal kidney structure is illustrated in Figure 2(a), depicting a kidney section from a normal DB/DB mouse. Glomeruli are numerous and distinct with patent capillaries, normal cellularity, and architecture, and their tubules are compact and of normal shape. In comparison, kidney sections from db-/db- mice (Figure 2(b)) exhibited severe disruption of kidney architecture, lesions involving all glomeruli and tubular atrophy. After AOE administration, especially at 500 mg/kg AOE, kidney structure was almost recovered (Figures 2(c)–2(e)). In addition, scores of renal injury (glomeruli and tubulointerstitial injury) in the db-/db-H_2_O group were higher than those in the DB/DB group (*P* = 2.82E − 10), while AOE treatment reduced scores of renal injury in a dose-dependent manner (*P* = 0.157 in db-/db-AOE100, *P* = 1.58E − 05 in db-/db-AOE300, and *P* = 2.94E in db-/db-AOE500 compared with db-/db-H_2_O mice) (Figures 2(f), 2(g)). Those results showed that 500 mg/kg AOE was the most effective at attenuating renal injury.

**Figure 3:**
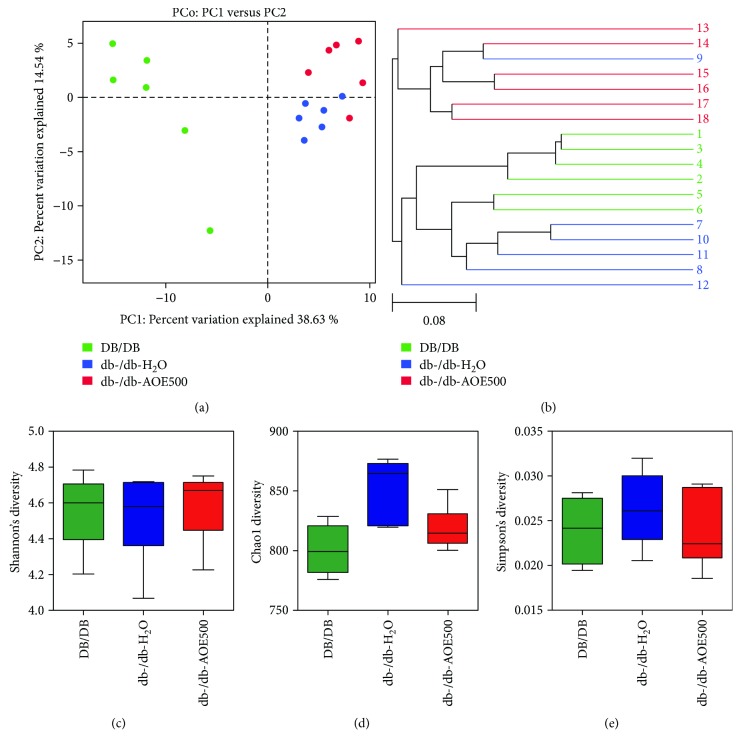
AOE modulated the structure and diversity of the gut microbiota. (a) Principal coordinate analysis (PCoA) and (b) sample clustering results of the unweighted UniFrac distances of microbial 16S rRNA sequences from the V3-V4 region in fecal contents at week 8. The alpha diversity analysis, including Shannon diversity (c), Chao1 diversity (d), and Simpson diversity (c). There were six mice in each group.

Thus, we assessed changes in the gut microbial community induced by AOE500. 16S ribosomal RNA gene from variable regions V3-V4 of the fecal samples from DB/DB, db-/db-H_2_O, and db-/db-AOE500 diet groups was sequenced by Illumina HiSeq/MiSeq platforms, and the results were presented as operational taxonomic units (OTUs) using a 97% homology cutoff value. The phylogenetic differences within the intestinal microbiota were assessed by principal coordinate analysis (PCoA) (Figure 3(a)). The db-/db-H_2_O mice had a distinct microbiota composition that clustered separately from the DB/DB mice. The db-/db-AOE500 mice were clearly distinct from the db-/db-H_2_O mice. Hierarchical clustering results were consistent with the previous PCoA analysis (Figure 3(b)). To assess intestinal microbial community structure, richness and evenness were calculated (Figures 3(c)–3(e)). Diversity measured by Shannon's richness index decreased slightly in the db-/db-H_2_O group compared with the DB/DB group. The db-/db-AOE500 mice had slightly increased values of Shannon's index compared with the db-/db-H_2_O group (Figure 3(c)). Diversity measured by Simpson's evenness index and CHAO1 diversity showed slightly increased values in the db-/db-H_2_O group compared with the DB/DB group. The values of Simpson's index and CHAO1 diversity in the db-/db-AOE500 group were slightly lower than those in the db-/db-H_2_O group (Figures 3(d), 3(e)).

**Figure 4:**
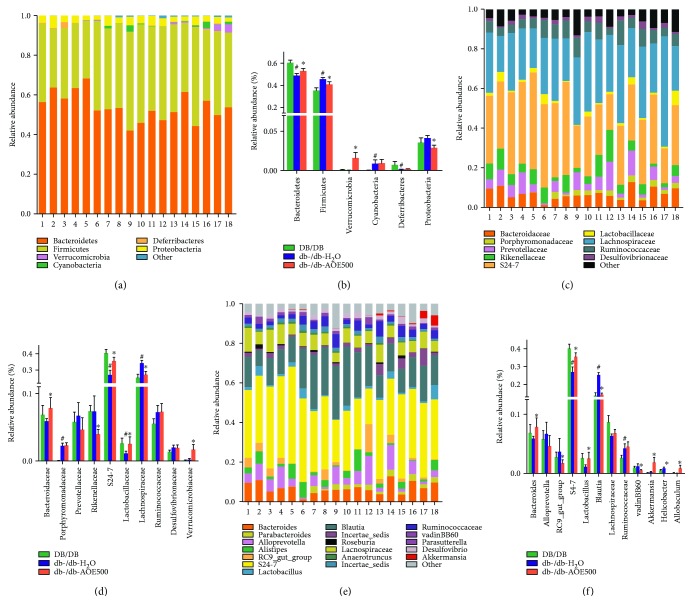
AOE modulated the composition of the gut microbiota. (a) Phylum-level, (b) family-level, and (c) genus-level taxonomic distributions of the microbial communities in fecal contents determined by next generation sequencing. Data represent the mean ± SD (*n* = 6). ^∗^*P* < 0.05 for db-/db-AOE group compared with db-/db-H_2_O, ^#^*P* < 0.05 for db-/db-H_2_O treated compared with DB/DB.

To assess specific changes in the gut microbiota, we compared the relative abundance of the predominant taxa identified from sequencing in the three diet groups (Figure 3). Significant differences in the composition of the gut microbiota were found at all taxonomic levels. At the phylum level, the db-/db-H_2_O-associated fecal microbiota community showed a dramatic increase in the relative abundance of Firmicutes (*P* = 0.00198, *Q* = 0.00396) and Cyanobacteria (*P* = 0.00014, *Q* = 0.00089) and a marked decrease in the relative abundance of Bacteroidetes (*P* = 0.00121, *Q* = 0.00362) compared with the DB/DB group (Figure 4(a)). Differing greatly from the db-/db-H2O group, the db-/db-AOE500-associated gut community had a trend toward an increase in the relative abundance of Bacteriodetes and decrease in Firmicutes (*P* = 0.04353, *Q* = 0.08706). On the other hand, the relative abundance of Verrucomicrobia (*P* = 0.01305, *Q* = 0.03916) showed a sharp increase in the db-/db-AOE500 mice (Figure 4(a)).

At the family level, the fecal microbiota was dominated by S24-7 followed by Lachnospiraceae in all groups. The db-/db-H_2_O-associated fecal microbiota community showed a dramatic increase in the relative abundance of Lachnospiraceae (*P* = 0.00912, *Q* = 0.03040) and Porphyromonadaceae (*P* = 0.00023, *Q* = 0.00237) and a marked decrease in the relative abundance of S24-7 (*P* = 0.00121, *Q* = 0.00605) and Lactobacillaceae (*P* = 0.03187, *Q* = 0.07969) (Figure 4(b)). The db-/db-AOE500 increased the relative abundance of Verrucomicrobiaceae by ninefold (*P* = 7.767E − 06, *Q* = 7.767E − 05), S24-7 33.3% (*P* = 0.00106, *Q* = 0.00533), Bacteroidaceae 34.4% (*P* = 0.00158, *Q* = 0.00528), and Lactobacillaceae 127.2% (*P* = 0.01991, *Q* = 0.03983) and decreased the relative abundance of Rikenellaceae 47.8% (0.00106, *Q* = 0.00533) and Lachnospiraceae 20.6% (*P* = 0.02484, *Q* = 0.04141) (Figure 4(b)).

**Figure 5:**
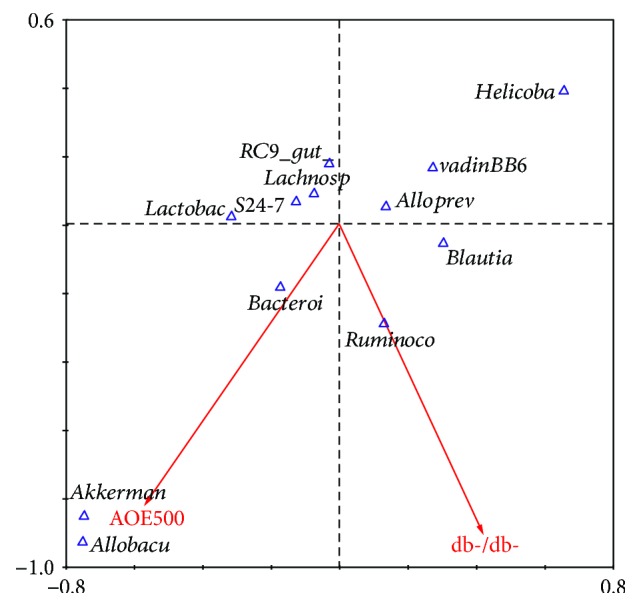
Canonical correspondence analysis (CCA) of bacterial community profiles from mice fecal samples. The length of each line represents the contribution of each variable to the bacterial community structure difference.

At the genus level, the db-/db-H_2_O-associated fecal microbiota community showed a dramatic increase in the relative abundance of *Blautia* (*P* = 0.00032, *Q* = 0.00387) and Ruminococcaceae (*P* = 0.00478, *Q* = 0.01914) and a marked decrease in the relative abundance of S24-7 (*P* < 0.05). The db-/db-AOE500 resulted in a sharp increase in *Akkermansia* and *Bacteroides* but a decrease in *Helicobacter* and *Blautia* (Figure 4(c)). The db-/db-AOE500 increased *Akkermansia* by ninefold (*P* = 3.45E − 05, *Q* = 0.00041) and *Bacteroides* 34% (*P* = 0.00544, *Q* = 0.01633), while it decreased *Helicobacter* 99.8% (*P* = 0.00248, *Q* = 0.00993) and *Blautia* 43.7% (*P* = 7.34E − 05, *Q* = 0.00044) (Figure 4(c)). For factors with a statistically significant fit, constrained canonical analysis (CCA) was performed at the genus levels. The results showed that *Akkermansia* might be a key target while db-/db- mice treated with AOE (Figure 5). Collectively, these results show that AOE500 modulates the gut microbiota of T2DM mice.

## 4. Discussion

Gut microbiota play an important role in the development of T2DM and metabolic disorders. Meanwhile, the composition of gut microbiota might be shaped by multiple diets, such as a high-fat diet and high-fiber diet. The alteration of gut microbiota diversity has a profound influence on host metabolism. Studies have shown that hyperglycemia results in overproduction of ROS, which impairs glucose-stimulated insulin secretion and induces insulin resistance [32, 33]. In our previous study, AOE exhibited antioxidative capacity in vitro and in vivo [23]. In addition, the blood glucose levels and urine albumin secretion were significantly reduced after AOE treatment. Antioxidative capacity may partly underlie mechanism in the treatment of diabetes with AOE; however, more information is required to determine the antidiabetic mechanism of AOE. In this study, we found that AOE not only has a hypoglycemic capacity but also modulates gut microbiota composition.

The mouse and human microbiota are similar at the division level, with Firmicutes and Bacteroidetes dominating. Many studies have shown that increased ratios of Firmicutes to Bacteroides are associated with a diabetic phenotype [34, 35]. Geurts et al. observed a significantly higher abundance of Firmicutes in db/db mice [36]. The early gut microbiota composition was found to be different between NOD mice that later in life were classified as diabetic or non-diabetic. Bacteroidetes act in favor of diabetes protection, whereas members of Firmicutes promote diabetes onset, regulatory imbalance, and IFN-gamma level in NOD mice [37]. Various studies have shown that dietary manipulation of gut microbiota may be a novel mechanism to delay diabetes onset [38]. Consumption of acidic water alters the gut microbiome and decreases the risk of diabetes [38]. Sardine-enriched diet interventions altered gut microbiota composition of drug-naive patients with T2DM [39]. Bamboo shoot fiber modulates the gut microbiota and improves host metabolism [10]. In agreement with these findings, we observed that the abundance of Firmicutes in db-/db-AOE mice markedly decreased compared with that in the db-/db-H_2_O mice and was much higher than that in the DB/DB mice. Meanwhile, the abundance of Bacteroidetes in db-/db-AOE500 mice markedly increased compared with that in the db-/db-H_2_O mice and was much less than that in the DB/DB mice. These results suggest that AOE selectively promoted growth of Bacteroidetes and inhibited growth of Firmicutes.

*Akkermansia* is a mucin-degrading bacterium that lives in the mucus layer of the intestine. There has also been growing interest in *Akkermansia* due to its association with health in animals and humans. *Akkermansia* prevented Western diet-induced inflammation in both the circulation and local atherosclerotic lesion [40, 41] and negatively mediated effects of IFN-*γ* on glucose tolerance [42]. Moreover, calorie restriction [43, 44], metformin treatment [15], or Roux-en-Y gastric bypass surgery [45] showed a higher abundance of *Akkermansia*. The increased amount of *Akkermansia* may be related to elevated GLP-1 secretion, elevated serum insulin, and improved insulin resistance [45]. In agreement with these findings, we found a sharp increase in the relative abundance of *Akkermansia* in db-/db- mice after AOE treatment. Therefore, alteration of *Akkermansia* levels might be important with regard to their functions.

Recent studies have demonstrated the possible existence of an association between infection with *Helicobacter pylori* and metabolic syndrome, such as in insulin resistance and DM [46, 47]. *Helicobacter* can influence the absorption of glucose and lipids in DM [48]. *Helicobacter* is associated with oxidative stress, and there is a cross-relation between *Helicobacter* and T2DM [49]. In the present study, the abundance of *Helicobacter* in db-/db-H_2_O mice markedly increased compared with that in the DB/DB mice. Meanwhile, AOE treatment significantly inhibited the growth of *Helicobacter*. The abundance of *Helicobacter* in db-/db-AOE500 was significantly less than that both in the db-/db-H_2_O and DB/DB mice. Thus, the suppression of *Helicobacter* will also help to elucidate the underlying mechanisms of AOE antidiabetic function. Since AOE was shown to play a role in controlling glucose level, future investigation using humanized mouse models and transfer experiments will provide more details about how AOE differentially modulates gut microbiota across species.

In conclusion, based on the results of the present study, we suggest that AOE treatment decreases blood glucose levels and plays a protective role in renal function in T2DM by modulating gut microbiota composition.

## References

[1] Shaw J. E., Sicree R. A., Zimmet P. Z. (2010). Global estimates of the prevalence of diabetes for 2010 and 2030. *Diabetes Research and Clinical Practice*.

[2] LeRoith D. (2008). Hyperglycemia, hypertension, and dyslipidemia in type 2 diabetes mellitus: goals for diabetes management. *Clinical Cornerstone*.

[3] Guo M., Ricardo S. D., Deane J. A., Shi M., Cullen-McEwen L., Bertram J. F. (2005). A stereological study of the renal glomerular vasculature in the db/db mouse model of diabetic nephropathy. *Journal of Anatomy*.

[4] Ussar S., Fujisaka S., Kahn C. R. (2016). Interactions between host genetics and gut microbiome in diabetes and metabolic syndrome. *Molecular Metabolism*.

[5] Barlow G. M., Yu A., Mathur R. (2015). Role of the gut microbiome in obesity and diabetes mellitus. *Nutrition in Clinical Practice*.

[6] Sasaki M., Ogasawara N., Funaki Y. (2013). Transglucosidase improves the gut microbiota profile of type 2 diabetes mellitus patients: a randomized double-blind, placebo-controlled study. *BMC Gastroenterology*.

[7] Graessler J., Qin Y., Zhong H. (2013). Metagenomic sequencing of the human gut microbiome before and after bariatric surgery in obese patients with type 2 diabetes: correlation with inflammatory and metabolic parameters. *The Pharmacogenomics Journal*.

[8] Wang L., Christophersen C. T., Sorich M. J., Gerber J. P., Angley M. T., Conlon M. A. (2011). Low relative abundances of the mucolytic bacterium Akkermansia muciniphila and Bifidobacterium spp. in feces of children with autism. *Applied and Environmental Microbiology*.

[9] Derrien M., Belzer C., de Vos W. M. (2017). *Akkermansia muciniphila* and its role in regulating host functions. *Microbial Pathogenesis*.

[10] Li X., Guo J., Ji K., Zhang P. (2016). Bamboo shoot fiber prevents obesity in mice by modulating the gut microbiota. *Scientific Reports*.

[11] Kang C., Zhang Y., Zhu X. (2016). Healthy subjects differentially respond to dietary capsaicin correlating with specific gut enterotypes. *The Journal of Clinical Endocrinology and Metabolism*.

[12] Anhê F. F., Roy D., Pilon G. (2015). A polyphenol-rich cranberry extract protects from diet-induced obesity, insulin resistance and intestinal inflammation in association with increased *Akkermansia* spp. population in the gut microbiota of mice. *Gut*.

[13] Roopchand D. E., Carmody R. N., Kuhn P. (2015). Dietary polyphenols promote growth of the gut bacterium Akkermansia muciniphila and attenuate high-fat diet-induced metabolic syndrome. *Diabetes*.

[14] Anhe F. F., Pilon G., Roy D., Desjardins Y., Levy E., Marette A. (2016). Triggering Akkermansia with dietary polyphenols: a new weapon to combat the metabolic syndrome?. *Gut Microbes*.

[15] Shin N. R., Lee J. C., Lee H. Y. (2014). An increase in the Akkermansia spp. population induced by metformin treatment improves glucose homeostasis in diet-induced obese mice. *Gut*.

[16] Shi S.-H., Zhao X., Liu B. (2014). The effects of sesquiterpenes-rich extract of *Alpinia oxyphylla* Miq. on amyloid-*β*-induced cognitive impairment and neuronal abnormalities in the cortex and hippocampus of mice. *Oxidative Medicine and Cellular Longevity*.

[17] Wang S., Zhao Y., Zhang J. (2015). Antidiarrheal effect of Alpinia oxyphylla Miq.(Zingiberaceae) in experimental mice and its possible mechanism of action. *Journal of Ethnopharmacology*.

[18] Zhang Q., Cui C., Chen C.-Q. (2015). Anti-proliferative and pro-apoptotic activities of Alpinia oxyphylla on HepG2 cells through ROS-mediated signaling pathway. *Journal of Ethnopharmacology*.

[19] An L. J., Guan S., Shi G. F., Bao Y. M., Duan Y. L., Jiang B. (2006). Protocatechuic acid from Alpinia oxyphylla against MPP+−induced neurotoxicity in PC12 cells. *Food and Chemical Toxicology*.

[20] Shi G.-F., An L.-J., Jiang B., Guan S., Bao Y.-M. (2006). Alpinia protocatechuic acid protects against oxidative damage in vitro and reduces oxidative stress in vivo. *Neuroscience Letters*.

[21] Wang H., Liu T.-Q., Guan S., Zhu Y.-X., Cui Z.-F. (2008). Protocatechuic acid from Alpinia oxyphylla promotes migration of human adipose tissue-derived stromal cells in vitro. *European Journal of Pharmacology*.

[22] Wang H., Liu T.-Q., Zhu Y.-X., Guan S., Ma X.-H., Cui Z.-F. (2009). Effect of protocatechuic acid from Alpinia oxyphylla on proliferation of human adipose tissue-derived stromal cells in vitro. *Molecular and Cellular Biochemistry*.

[23] Xie Y., Xiao M., Li D. (2017). Anti-diabetic effect of Alpinia oxyphylla extract on 57BL/KsJ db−/db- mice. *Experimental and Therapeutic Medicine*.

[24] Du G., Xiao M., Zhang X. (2017). Alpinia oxyphylla Miq. extract changes miRNA expression profiles in db−/db- mouse kidney. *Biological Research*.

[25] Nicklas W., Baneux P., Boot R. (2002). Recommendations for the health monitoring of rodent and rabbit colonies in breeding and experimental units. *Laboratory Animals*.

[26] Lott J. A., Turner K. (1975). Evaluation of Trinder's glucose oxidase method for measuring glucose in serum and urine. *Clinical Chemistry*.

[27] Katafuchi R., Kiyoshi Y., Oh Y. (1998). Glomerular score as a prognosticator in IgA nephropathy: its usefulness and limitation. *Clinical Nephrology*.

[28] Magoc T., Salzberg S. L. (2011). FLASH: fast length adjustment of short reads to improve genome assemblies. *Bioinformatics*.

[29] DeSantis T. Z., Hugenholtz P., Larsen N. (2006). Greengenes, a chimera-checked 16S rRNA gene database and workbench compatible with ARB. *Applied and Environmental Microbiology*.

[30] Edgar R. C. (2013). UPARSE: highly accurate OTU sequences from microbial amplicon reads. *Nature Methods*.

[31] Braak T., Cajo J. F., Smilauer P. CANOCO reference manual and CanoDraw for Windows user's guide: software for canonical community ordination (version 4.5). http://www.canoco.com.

[32] Rolo A. P., Palmeira C. M. (2006). Diabetes and mitochondrial function: role of hyperglycemia and oxidative stress. *Toxicology and Applied Pharmacology*.

[33] Ihara Y., Toyokuni S., Uchida K. (1999). Hyperglycemia causes oxidative stress in pancreatic beta-cells of GK rats, a model of type 2 diabetes. *Diabetes*.

[34] Larsen N., Vogensen F. K., van den Berg F. W. J. (2010). Gut microbiota in human adults with type 2 diabetes differs from non-diabetic adults. *PLoS One*.

[35] Murri M., Leiva I., Gomez-Zumaquero J. M. (2013). Gut microbiota in children with type 1 diabetes differs from that in healthy children: a case-control study. *BMC Medicine*.

[36] Geurts L., Lazarevic V., Derrien M. (2011). Altered gut microbiota and endocannabinoid system tone in obese and diabetic leptin-resistant mice: impact on apelin regulation in adipose tissue. *Frontiers in Microbiology*.

[37] Krych L., Nielsen D. S., Hansen A. K., Hansen C. H. (2015). Gut microbial markers are associated with diabetes onset, regulatory imbalance, and IFN-*γ* level in NOD mice. *Gut Microbes*.

[38] Wolf K. J., Daft J. G., Tanner S. M., Hartmann R., Khafipour E., Lorenz R. G. (2014). Consumption of acidic water alters the gut microbiome and decreases the risk of diabetes in NOD mice. *Journal of Histochemistry and Cytochemistry*.

[39] Balfegò M., Canivell S., Hanzu F. A. (2016). Effects of sardine-enriched diet on metabolic control, inflammation and gut microbiota in drug-naïve patients with type 2 diabetes: a pilot randomized trial. *Lipids in Health and Disease*.

[40] Zhao S., Liu W., Wang J. (2017). *Akkermansia muciniphila* improves metabolic profiles by reducing inflammation in chow diet-fed mice. *Journal of Molecular Endocrinology*.

[41] Li J., Lin S., Vanhoutte P. M., Woo C. W., Xu A. (2016). Akkermansia muciniphila protects against atherosclerosis by preventing metabolic endotoxemia-induced inflammation in Apoe−/− mice. *Circulation*.

[42] Greer R. L., Dong X., Moraes A. C. F. (2016). *Akkermansia muciniphila* mediates negative effects of IFN*γ* on glucose metabolism. *Nature Communications*.

[43] Dao M. C., Everard A., Aron-Wisnewsky J. (2016). Akkermansia muciniphila and improved metabolic health during a dietary intervention in obesity: relationship with gut microbiome richness and ecology. *Gut*.

[44] Schneeberger M., Everard A., Gomez-Valades A. G. (2015). Akkermansia muciniphila inversely correlates with the onset of inflammation, altered adipose tissue metabolism and metabolic disorders during obesity in mice. *Scientific Reports*.

[45] Yan M., Song M. M., Bai R. X., Cheng S., Yan W. M. (2016). Effect of Roux-en-Y gastric bypass surgery on intestinal Akkermansia muciniphila. *World Journal of Gastrointestinal Surgery*.

[46] Malamug L. R., Karnchanasorn R., Samoa R., Chiu K. C. (2014). The role of *Helicobacter pylori* seropositivity in insulin sensitivity, beta cell function, and abnormal glucose tolerance. *Scientifica*.

[47] Kozyrieva T., Kolesnikova E., Shut I. (2016). Correlation of Helicobacter pylori infection with development of cardiovascular risk in patients with coronary heart disease in association with type 2 diabetes mellitus. *Georgian Medical News*.

[48] Tsang K., Lam S. (1999). Extragastroduodenal conditions associated with Helicobacter pylori infection. *Hong Kong Medical Journal*.

[49] Nasif W. A., Mukhtar M. H., Nour Eldein M. M., Ashgar S. S. (2016). Oxidative DNA damage and oxidized low density lipoprotein in type II diabetes mellitus among patients with Helicobacter pylori infection. *Diabetology & Metabolic Syndrome*.

